# Auditory Verbal Hallucinations and Brain Dysconnectivity in the Perisylvian Language Network: A Multimodal Investigation

**DOI:** 10.1093/schbul/sbt172

**Published:** 2013-12-22

**Authors:** Stefania Benetti, William Pettersson-Yeo, Paul Allen, Marco Catani, Steven Williams, Alessio Barsaglini, Lana M. Kambeitz-Ilankovic, Philip McGuire, Andrea Mechelli

**Affiliations:** ^1^Department of Psychosis Studies, King’s College Health Partners, King’s College London, London, UK;; ^2^Centre for Mind/Brain Sciences, University of Trento, Trento, Italy;; ^3^Department of Forensic and Neurodevelopmental Science, King’s College Health Partners, King’s College London, London, UK;; ^4^Department of Neuroimaging, King’s College Health Partners, King’s College London, London, UK;; ^5^Department of General Psychology, Università degli Studi di Padova, Padova, Italy;; ^6^Department of Psychiatry, Ludwig-Maximilians University, Munich, Germany

**Keywords:** connectivity, auditory verbal hallucinations, psychosis

## Abstract

Neuroimaging studies of schizophrenia have indicated that the development of auditory verbal hallucinations (AVHs) is associated with altered structural and functional connectivity within the perisylvian language network. However, these studies focussed mainly on either structural or functional alterations in patients with chronic schizophrenia. Therefore, they were unable to examine the relationship between the 2 types of measures and could not establish whether the observed alterations would be expressed in the early stage of the illness.

We used diffusion tensor imaging and functional magnetic resonance imaging to examine white matter integrity and functional connectivity within the left perisylvian language network of 46 individuals with an at risk mental state for psychosis or a first episode of the illness, including 28 who had developed AVH group and 18 who had not (nonauditory verbal hallucination [nAVH] group), and 22 healthy controls. Inferences were made at *P* < .05 (corrected). The nAVH group relative to healthy controls showed a reduction of both white matter integrity and functional connectivity as well as a disruption of the normal structure−function relationship along the fronto-temporal pathway. For all measures, the AVH group showed intermediate values between healthy controls and the nAVH group. These findings seem to suggest that, in the early stage of the disorder, a significant impairment of fronto-temporal connectivity is evident in patients who do not experience AVHs. This is consistent with the hypothesis that, whilst mild disruption of connectivity might still enable the emergence of AVHs, more severe alterations may prevent the occurrence of the hallucinatory experience.

## Introduction

Auditory verbal hallucinations (AVHs) are one of the most debilitating symptoms of schizophrenia.^1^ Over the past 3 decades, neuroimaging studies of AVHs have revealed structural and functional alterations in frontal and temporal brain areas that are part of the language network (see reviews for detail^2,3^). More recently, these studies have provided evidence for a disruption of fronto-temporal interactions, consistent with the notion that the core symptoms of schizophrenia cannot be accounted for solely in terms of regional alterations but might be best explained in terms of dysconnectivity.^4,5^ Specifically, diffusion tensor imaging (DTI) studies have revealed alterations of white matter integrity in the main bundle connecting perisylvian language regions, namely the arcuate fasciculus (AF),^6–10^ whereas functional magnetic resonance imaging (fMRI) and electroencephalography (EEG) studies have demonstrated altered functional connectivity between temporoparietal and inferior frontal regions.^11–13^

At present, it is difficult to integrate these results into a coherent theoretical framework for a number of reasons. Firstly, the direction of the findings has been inconsistent. For instance, DTI studies have reported both decreased^6–9^ and increased^14–17^ fractional anisotropy (FA) values in patients with AVHs relative to healthy controls; similarly, fMRI and EEG studies have shown both decreases^12,18^ and increases^19^ in frontotemporal interactions. These inconsistencies might at least in part be explained by the relatively small sample size (*n* < 15) of the majority of existing studies. Secondly, the neurobiology of AVHs has mainly been investigated in patients with chronic schizophrenia; thus it has not been possible to examine whether the observed alterations are specifically associated with the development of AVHs or reflect the effects of prolonged exposure to the hallucinatory experience. De Weijer and colleagues^9^ have recently investigated white matter integrity of the AF in nonpsychotic hallucinators and chronic patients with AVHs, and have reported FA reductions in the latter but not the former group. This appears to suggest that FA alterations of the AF cannot be explained in terms of prolonged exposure to AVHs but may reflect other disease processes in schizophrenia; this conclusion however is tentative because a group of chronic patients without AVHs was not included in the study. Thirdly, because all previous studies of AVHs have focussed on either structural or functional alterations, it has not been possible to examine the relationship between the 2 types of measure. Thus it is unclear whether the development of AVHs is also associated with a disruption of the normal structure−function relationship.

We aimed to examine for the first time both structural and functional connectivity within the perisylvian language network in a larger cohort of individuals who shared vulnerability to psychosis, because they presented with an At Risk Mental State (ARMS) or a First Episode of Psychosis (FEP), but differed in terms of AVHs symptomatology. Specifically, we employed DTI and fMRI to estimate the white matter integrity and functional connectivity along the 3 segments of the left AF that are thought to connect frontal, temporal and parietal regions of the perisylvian language network.^20^ The acquisition of both DTI and fMRI data within the same cohort allowed us to examine the relationship between white matter integrity and functional connectivity as a function of AVHs symptomatology. Based on the results of previous studies on chronic patients^6−8,11,12^ we expected that the development of AVHs would be associated with altered white matter integrity of the AF fibers originating in the posterior temporal cortex as well as altered inter-regional coupling between temporal and frontal regions as measured with fMRI. In addition, based on current neurobiological models of dysconnectivity in schizophrenia,^4,5^ we hypothesized a disruption of the normal structure−function relationship within the left perisylvian language network as a function of AVHs symptomatology.

## Methods

### Subject Recruitment and Assessment

This study was approved by the local Research Ethics Committee. Participants gave written informed consent after a full description of the aims and design of the study. Forty-six individuals with vulnerability to psychosis were recruited from early intervention services within the South London and Maudsley National Health Foundation Trust; inclusion criteria included (1) meeting the Personal Assessment and Crisis Evaluation (PACE) criteria for the At Risk Mental State (ARMS)^21^ or (2) having recently presented with a FEP. This group was further subdivided into those with and without AVHs symptomatology. This was based on the scores of the Positive And Negative Syndrome Scale (PANSS)^22^ at the time of scanning, as well as detailed information regarding past symptomatology acquired through patient interview and examination of patient’s medical records. In particular, participants were assigned to the AVH group if they scored ≥3 on item P3 of the PANSS: “One or two clearly formed but infrequent AVHs, or else a number of vague abnormal perceptions which do not result in distortions of thinking or behavior” either (1) at the time of scanning or (2) prior to the time of scanning and at any time before or after referral to an early intervention service. Twenty-eight (14 females, 14 males; 15 ARMS, 13 FEP) subjects were included in the hallucinator (AVH) group while the remaining 18 subjects (12 males, 6 females; 5 ARMS, 13 FEP) were included in the nonhallucinator (nAVH) group. The PANSS was used to assess the presence and severity of clinical symptoms at the time of scanning, whereas the Psychotic Symptoms Rating Scales (PSYRATS) were used to further assess the severity of AVHs and delusions. To further characterize the AVH and nAVH group in terms of symptoms, we estimated the severity of disorganization symptoms, excitement and emotional distress as suggested by van der Gaag et al.^23,24^ In addition, 22 healthy volunteers were recruited by advertisement from the local community. Additional exclusion criteria included (1) a current or past history of psychiatric illness and (2) the presence of psychosis in first-degree relatives. The Prodomal Questionnaire^25^ was used to confirm the absence of any psychotic syndromes in healthy volunteers.

### Data Acquisition

All subjects underwent both DTI and fMRI scanning on a 3.0 T GE Signa system (GE Medical Systems). For DTI, each volume was acquired with 40 mT/m gradients and using a cardiac gated acquisition sequence from 60 contiguous near-axial slice locations providing isotropic resolution (2.4 × 2.4 × 2.4 mm) with a FOV equal to 30.7 and a matrix size of 128 × 128. Full details of the acquisition sequence are provided by Jones and colleagues.^26^ For fMRI, a total of 600 image volumes were acquired for each subject in two runs (300 for initiation and 300 for suppression), each lasting 10 min (TR of 2 s, flip angle of 70°, TE of 30 ms, slice thickness of 3 mm, interslice gap of 0.3 mm and field of view 240 mm). Thirty axial slices parallel to the AC-PC line were acquired with an image matrix of 64 × 64 (Read × Phase) providing whole-brain coverage.

### Functional Magnetic Resonance Imaging Experimental Task

Functional MRI data were collected while participants performed an adapted version of the Hayling Sentence Completion Task (HSCT)^27^; this cognitive task was chosen because it had been shown to elicit robust activation of the perisylvian language network in previous studies.^28,29^ A more detailed description of the experimental task is available elsewhere.^28^ In brief, 80 sentence stems were selected from those provided by Arcuri and colleagues^30^ and Bloom and Fisher^31^ on the basis of being associated with a high (>.9) or low (<.5) probability of completing the sentence with one particular word and arranged into block of 5 stems. Sentence stems consisted of 5, 6, or 7 words and were assigned to either a response Initiation condition, in which a semantically congruent response was required (eg, He posted the letter without a “STAMP”), or a response Suppression condition, in which participants were required to provide a semantically noncongruent response (eg, The boy went to an expensive “Shoe”). In addition, the experimental paradigm comprised of a control condition, here referred to as Repetition, in which participants were presented with the word “REST” and were instructed to read it overtly. The 40 sentence stems assigned to each congruency condition were arranged into blocks, which contained 5 sentence stems each. The 2 conditions (ie, Initiation and Suppression) were presented in 2 separate acquisition sessions. Within each condition, the level of constraint was alternated between each block in an ABABABAB design.

After the acquisition of the DTI and fMRI images, participants of both the AVH and nAVH group were inquired as to whether they had experienced AVHs at any time during the scanning; none were reported.

## Data Processing and Analysis

### Diffusion Tensor Imaging

#### Virtual Tractography.

**Fig. 1.**
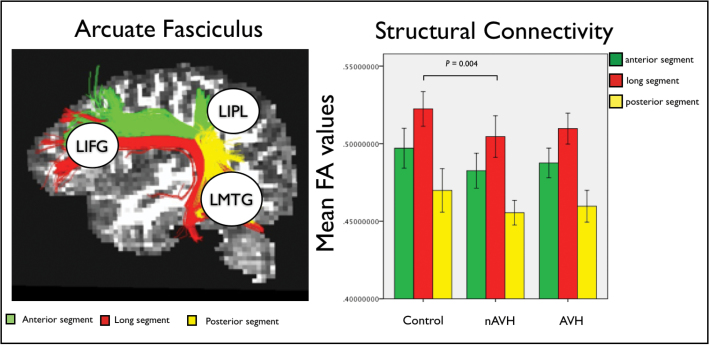
The 3 segments of the arcuate fasciculus (AF) connecting left inferior frontal gyrus (LIFG), left middle temporal gyrus (LMTG), and left inferior parietal lobe (LIPL) within the perisylvian language network (left). Group-specific mean fractional anisotropy (FA) values for each segment of the AF and significant group difference between healthy controls and the nonauditory verbal hallucinations (nAVH) group (right). Error bars indicate 95% CI.

A detailed description of the method used to derive FA values has been published previously.^26,32,33^ In brief, data were first pre-processed correcting for eddy current distortions and head motion. For each subject the b-matrix was reoriented to provide a more accurate estimate of tensor orientations.^34^ Diffusion tensors were estimated using RESTORE^35^ and whole brain tractography was performed using a b-spline interpolation of the DTI field and Euler integration^33^ to propagate streamlines following the directions of the principal eigenvector with a step size of 0.5mm. Tractography was started in all brain voxels with FA > 0.2. Streamlines were tracked until the FA of the tensor was above an FA threshold of 0.2 or the curvature (ie, the angle between 2 consecutive steps) was less than 30°. A virtual dissection of the left AF and its 3 segments connecting frontal, parietal and temporal regions was performed following the procedure described in Catani et al.^20^ A 2 regions of interest (ROIs) approach was used to dissect separately the medial and lateral segments of this associative frontotemporal bundle (figure 1, left).

#### Statistical Analysis of Fractional Anisotropy.

Mean FA values were analyzed in SPSS (version 19.0, IBM Comp. & SPSS Inc., 2010). A multifactorial general linear model analysis (ie, repeated measure) was implemented with segment as within-subjects factors and group as a between-subject factor. Significant group differences (at *P *< .05) were characterized further by performing 3 independent one-way ANOVAs using a Bonferroni-corrected threshold of *P* *= *.016 (ie, 0.05/3 connections). When detected, group differences in a specific tract were further investigated using post hoc 2 sample *t* tests and a Bonferroni-corrected threshold of *P* = .016 (ie, 0.05/3 groups). Age and gender were entered in each analysis as covariates of no interest in order to minimize the potential impact of these variables on the results.

### Functional Magnetic Resonance Imaging

#### Preprocessing.

Preprocessing and statistical analysis of functional data were performed using SPM8 software (http//www.fil.ion.ucl.ac.uk/spm), running in Matlab 10 (Matworks Inc). After visual inspection for artefacts, the images were realigned to the first volume of the first run and resliced with sync interpolation. The realigned images were spatially normalized to a standard MNI-305 EPI template^36^ using nonlinear basis functions and smoothed with a 6-mm FWHM isotropic Gaussian kernel.

#### Statistical Analysis of Regional Responses.

The standard voxel-wise statistical analysis of regional responses focussed on correct trails only in order to control for the potential impact of group differences in task performance on the results. The parameters estimates were calculated for each condition and contrast images were computed for each comparison of interest (ie, Initiation vs Repetition; Suppression vs Repetition; and Initiation vs Suppression). The subject-specific contrast images were then entered into a second-level random effects analysis to allow inference at group level, with age and gender defined as covariates of no interest to minimize their potential impact on the results. Inferences were made using a statistical threshold of *P* < .05 after FWE correction for multiple comparisons at voxel level across the whole brain and an extent threshold of 5 voxels.

#### Statistical Analysis of Functional Connectivity.

**Fig. 2.**
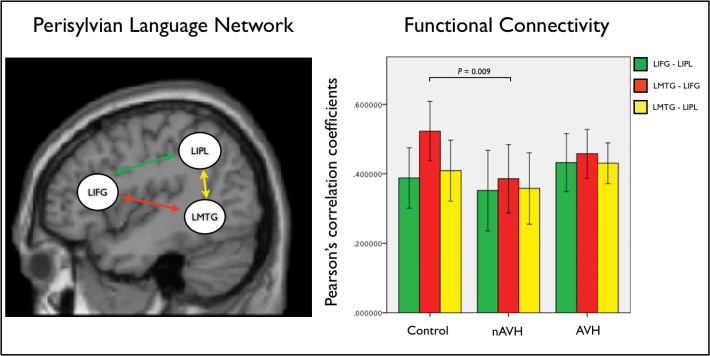
The perisylvian language network and functional connections investigated in this study (left). Group-specific mean Pearson’s correlation coefficients between time series in the regions of interest within the perisylvian language network and significant group difference between healthy controls and the nAVH group (right). Error bars indicate 95% CI.

The time series were extracted from 3 ROIs: the left inferior frontal gyrus (LIFG), the left middle temporal gyrus (LMTG), and the left inferior parietal lobule (LIPL). These regions were chosen because they play a critical role in language processing^37,38^ and are part of the perysilvian language network that is connected through the AF^20^ (figure 2, left). In order to ensure comparability across subjects and groups, the extraction of time series had to meet a combination of anatomical and functional criteria.^39^ Anatomically, the search for each subject-specific local maximum was constrained within the same correspondent cortical area, as defined by the PickAtlas toolbox.^40^ Functionally, the principal eigenvariates were extracted to summarize regional responses in 12mm spheres centered on each ROI. To account for individual differences, the location of these regions was based upon the local maxima of the subject-specific statistical parametric maps, defined as the nearest (within 10mm) of the group maxima. The resulting mean coordinates in Montreal Neurological Institute (MNI) space for the LIFG, LMTG and LIPL were [−40, 22, −6], [−58, −40, 2], and [−47, −59, 40], respectively. The subject- specific time series extracted from each region of interest were then multiplied by a vector encoding all correct trail time onsets in order to control for the potential impact of group difference in task performance on the results.^41^ For each subject, a Pearson’s correlation coefficient was calculated between each ROI using the extracted time series. The subject-specific correlation coefficients were then entered into a repeated measure ANOVA in order to identify significant differences (at *P* < .05) among the 3 experimental groups. Significant group differences were further characterized by implementing a number of post hoc analyses consistent with the methodological approach applied to the DTI data (see section above). Again, age and gender were defined as covariates of no interest in all analyses in order to minimize their potential impact on the results.

## Results

### Demographic and Clinical Characteristics

There were no significant group differences in age, handedness or premorbid IQ. With the exception of AVHs, there were no significant differences between the AVH and nAVH groups in terms of clinical characteristics with the exception of disorganization symptoms that were more pronounced in the nAVH group (table 1).

**Table 1.** Demographic and Clinical Characteristics of the 68 Participants

	Healthy Controls (*n* = 22)	No Proneness to AVHs (*n* =18)	Proneness to AVHs (*n* = 28)	Statistics
Age (SD)	24.05 (4.2)	25.06 (5.3)	24.07 (4.99)	*F* = 0.227, *P* = .751
Male/female	11M:11F	13M:5F	14M:14F	χ^2^ *=*2.281, *P* =.319
Premorbid IQ (SD)	108.1 (8.5)	101.05 (11.6)	103.14 (12.1)	*F* = 2.318, *P* = .107
Antipsychotic (mean CPZ equivalent)	NA	197.17 (247.7)	90.17 (113.1)	*t =*1.993, *P* = .052
Mean time from referral (days)	NA	267.96 (246.54)	166.06 (121.08)	*t* = 1.627, *P* =.111
Hand laterality index (4 = right; −4 = left)	3.09 (1.84)	3.11 (1.52)	2.89 (2.55)	*F* = 0.80, *P* = .923
Psychopathology scores, mean (SD)				
PANSS total	NA	54.94 (10.3)	53.5 (13.8)	*t* = 0.380, *P* = .706
PANSS positive	NA	12.83 (4.4)	13.57 (4.5)	*t* = 0.527, *P* = .601
PANSS negative	NA	14.72 (4.4)	13.57 (4.5)	*t* = 0.838, *P* = .406
PANSS hallucinations	NA	1.27 (0.4)	4.21 (1.06)	*t* = 11.00, *P* < .01
PANSS delusions	NA	3.11 (1.6)	2.75 (1.2)	*t* = 0.831, *P* = .410
Disorganization symptom^a^	NA	18.16 (5.0)	14.85 (4.97)	*t* = 2.19, *P* = .033
Excitement^a^	NA	11.72 (2.08)	12.10 (3.79)	*t* = 0.393, *P* = .696
Emotional distress^a^	NA	13.55 (2.47)	15.28 (4.60)	*t* = 1.46, *P = .151*
PSYRATS hallucinations	NA	0.16 (0.70)	16.25 (12.18)	*t* = 5.57, *P* < .001
PSYRATS delusions	NA	10.11 (7.03)	9.78 (7.10)	*t* = 0.15, *P* = .851

*Note*: AVHs, auditory verbal hallucinations; CPZ, chlorpromazine hydrochloride; PANSS, Positive And Negative Syndrome Scale; PSYRATS, Psychotic Symptom Rating Scales.

^a^From 5-factor model of PANSS by van deer Gaag et al.^23,24^

### Behavioral Performance (HSCT)

A repeated-measures ANOVA revealed a main effect of group (*F* = 7.469, *P *= .001, df = 1,62, pes = 0.194); post hoc *t* tests indicated that both the (AVH *t* = 3.65, df = 1,46, *P* = .001) and nAVH (*t *= 3.67, df = 1,37, *P* = .001) groups made more errors than the healthy controls but did not differ between them (*P* = .551).

### Diffusion Tensor Imaging

Repeated-measure analysis of FA measurements extracted from each single segment of the bilateral AF revealed a significant effect of group (*F* = 3.729, *P* = .031; table 2; figure 1, right). Post hoc analyses revealed a significant reduction of FA in the left long segment in the nAVH group compared to healthy controls (*F* = 9.565, *P* = .004). The AVH group showed intermediate values of this segment compared to the nAVH group and healthy controls, and did not differ significantly from either group using our Bonferroni-corrected threshold of *P* ≤ .016. When the nAVH and AVH groups were combined, there was not significant FA difference in the left long segment relative to healthy controls (*F* = 5.49, *P* > .016).

**Table 2.** Functional Connectivity Between Our 3 ROIs (Top Left) and Mean FA Values for Each Segment of the AF (Top Right)

Brain Region Connections	Functional Connectivity			AF Segments	Fractional Anisotropy		
	Controls	nAVH	AVH		Controls	nAVH	AVH
LIFG-LIPL	0.38 (0.18)	0.35 (0.23)	0.43 (0.21)	Anterior	0.49 (0.02)	0.48 (0.02)	0.48 (0.02)
LMTG-LIFG	0.52 (0.18)	0.38 (0.19)	0.46 (0.17)	Long	0.52 (0.02)	0.50 (0.02)	0.51 (0.02)
LMTG-LIPL	0.41 (0.18)	0.36 (0.20)	0.43 (0.17)	Posterior	0.47 (0.02)	0.46 (0.01)	0.46 (0.02)
Functional connectivity and fractional anisotropy relationship: Pearson’s correlation coefficients							
Connection		Controls		nAVH		AVH	
LMTG-LIFG and long segment AF		*R* = −.517, *P* = .023*		*R* = .144, *P =* .580*		*R* = −.306, *P =* .129	

*Note*: Standard deviation values are reported in brackets. Pearson’s correlation coefficients between frontotemporal functional connectivity and fractional anisotropy measures (bottom). AF, arcuate fasciculus; AVH, proneness to AVH; LIFG, left inferior frontal gyrus; LMTG, left middle temporal gyrus; LIPL, left inferior parietal lobe; nAVH, no proneness to AVH.

*Difference between controls and nAVH significant at *P* = .026.

### Functional Magnetic Resonance Imaging

#### Regional Activation.

Increased BOLD response across all task conditions (response Initiation and Suppression) compared to Repetition was observed in a frontotemporal network of regions including the left middle and dorsal superior frontal gyrus, the bilateral ventrolateral inferior frontal and lateral middle temporal gyri (see supplementary material for a detailed description). There were neither significant main effects of group nor significant group by task interactions (*P* > .05, FWE corrected).

#### Functional Connectivity.

Within each experimental group, a significant positive functional connectivity was observed between the LIFG and LIPL, the LMTG and LIFG, and the LMTG and LIPL, respectively. When comparing the strength of functional connections between groups, a significant reduction between the LMTG and LIFG was observed in the nAVH group compared to healthy controls (*F* = 7.764, *P* = .009) (table 2; figure 2, right). The AVH group showed intermediate values for functional connection compared to the nAVH group and healthy controls, and did not differ significantly from either group (*P* > .016). When the nAVH and AVH groups were combined, there was no significant difference relative to healthy controls (*P* > .016).

### Structure−Function Relationship

**Fig. 3.**
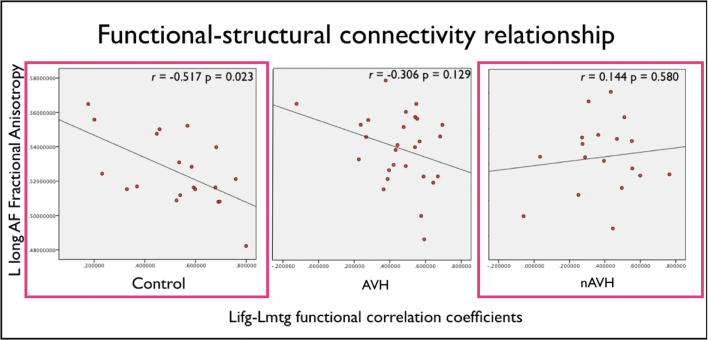
Scatterplots and correlation slopes showing a significant negative association between functional connectivity and fractional anisotropy (FA) values along the left frontotemporal pathway in healthy controls. Plotted values are adjusted for age. The colored frameworks indicate a significant difference between healthy controls and the nAVH group at *P* = .026 (for a color version, see this figure online).

Finally, we investigated the association between the white matter integrity of the long segment of the AF and the functional connectivity between the LMTG and LIFG since these measures had shown significant differences in the nAVH group relative to healthy controls (table 2; figure 3). We observed a significant negative correlation between these measures in the control group (*R *= −0.517, *P* = .023) but not in the nAVH group (*R *= 0.144, *P *= .580). Furthermore, Fisher’s *r-to-z* transformation^42^ was used to convert the groups-specific FC-FA correlation coefficients in group-specific *z*-scores, so that classical parametric statistical tests could be applied to contrast the 2 groups. This procedure revealed a significant difference between the healthy controls and the nAVH group (*t =* 1.91, *P* = .026). The AVH group showed intermediate correlation values (*R* = −0.306, *P *= .129) compared to the nAVH group and healthy controls, and did not differ significantly from either group. Finally, when the nAVH and AVH groups were combined, there was no significant difference relative to healthy controls.

#### Impact of Medication.

Although the AVH and nAVH groups did not differ in terms of medication dose (table 1), we explored the potential impact of medication on our results by testing for an association between chlorpromazine mean dose (mg/day) and the FA values of the long segment of the AF and the functional connectivity values between the LMTG and LIFG, respectively. Medication dose was not significantly associated with frontotemporal structural (*r* = 0.13, *P* = .18) or functional (*r* = −0.16, *P* = .14) connectivity values, suggesting that medication was not a likely explanation for our results.

#### Impact of Disorganization Symptoms.

Because nAVH individuals had higher scores on the disorganization scale compared to the AVH group (table 1), we explored the potential impact of this difference on our results by testing for an association between disorganization symptoms and measures of structural and functional connectivity along the left frontotemporal pathway in the nAVH group. We found no evidence that the structural or functional alterations observed along this pathway were associated with severity of disorganization symptoms (FA: *R* = −0.354, *P *= .150; FC: *R* = −0.043, *P* = .867).

## Discussion

The aim of this study was to examine structural and functional connectivity within the perisylvian language network as a function of AVHs symptomatology. Critically, our AVH and nAVH groups only differed in terms of hallucinatory experience, and were comparable in terms of all other clinical characteristics. We report similar patterns of structural and functional alteration, comprising a reduction in white matter integrity of the long medial segment of the AF and a reduction in functional connectivity between the LIFG and the LMTG, in the nAVH group relative to healthy controls. For both modalities, the AVH group showed intermediate values compared to the nAVH group and healthy controls, and did not differ significantly from either group. In addition, there was a negative correlation between the white matter integrity of the long medial segment of the AF and the functional connectivity between the LIFG and the LMTG in healthy controls that was not observed in the nAVH group. In contrast, no differences in regional activation were detected between groups consistent with the notion that the core symptoms of schizophrenia may be better explained in terms of dysconnectivity rather than localized deficits.^4,5^

The observation of pronounced connectivity alterations in the nAVH but not the AVH group may appear inconsistent with previous studies reporting perturbed structural frontotemporal pathways,^6–9^ while there is less consistent evidence of altered frontotemporal functional coupling in individuals with AVHs relative to healthy controls.^11–13^ Indeed, as discussed in the Introduction, our observation of unimpaired structural and functional connectivity in individuals with AVHs is not an unprecedented finding. For instance, there are a number of reports of preserved or increased frontotemporal structural^15,16,43^,^44^ and functional^19^ connectivity in patients with AVHs relative to healthy controls as well as a positive correlation between severity of AVHs and white matter integrity of the AF.^19^ Relevantly, de Weijer and colleagues^9^ have recently showed only mild increases in magnetization transfer ratio in the AF of nonpsychotic individuals with AVHs and further FA reductions only in chronic schizophrenia patients. Taken collectively, this pattern of results is consistent with the notion that a severe impairment of frontotemporal connectivity is not necessarily associated with the vulnerability to and the emergence of AVHs. Instead, a certain degree of preservation of these pathways may be required for integrating disorganized percepts into AVHs. For instance, Maher^45^ proposed that psychotic symptoms emerge as the brain attempts to integrate disorganized neural activity into a coherent and realistic, although pathological, framework. More recently, Whitford and colleagues^44^ have suggested that such attempt to integrate disorganized neural activity, which leads to the emergence of psychotic symptoms, may occur in the context of limited interregional desynchronization. Our findings seem to provide support to this intriguing hypothesis. It should be noted that for the purpose of the present study we focused on the structural and functional connectivity of the perisylvian language pathways; it is therefore possible that individuals with vulnerability to AVHs would present with structural and/or functional alterations in other pathways that were not the focus of the present study. Interestingly, a model for hallucinations beyond the auditory and linguistic domain has recently been proposed suggesting that the emergence of AVHs might be better characterized in terms of spatial and temporal instabilities of the Default Model Network.^46^

The macro-scale alterations in structural and functional connectivity detected in the present investigation are typically explained in terms of aberrant developmental wiring, synaptic plasticity or a combination of the two.^4,47^ One influential neurobiological model of schizophrenia suggests that macro-scale dysconnectivity results from aberrant *N*-methyl-d-aspartate glutamatergic receptor mediated synaptic plasticity, itself a consequence of abnormal regulation by neuromodulatory transmitters including dopamine, serotonin, and acetylcholine. This aberrant synaptic plasticity in turn results in alterations in gross brain structure and function leading to structural dysconnectivity between spatially remote regions.^4^ Our results, particularly the observation of similar patterns of structural and functional dysconnectivity in the nAVHs group, are consistent with this neurobiological model and the notion that aberrant *N*-methyl-d-aspartate glutamatergic receptor mediated synaptic plasticity may be critically associated with the emergence of specific symptoms.^4^ However, further integrative work would be required to better understand the relationship between macro-scale alterations in structural and functional connectivity and the underlying changes at synaptic and cellular level. Moreover, a longitudinal approach would be required to examine whether the relationship between frontotemporal connectivity and vulnerability to AVHs differs between those individuals at high-risk developing or not psychosis, or between those patients with a FEP who do and do not develop chronicity.

The present investigation has a number of limitations that need to be considered. Firstly, the FEP patients included in this work had been ill for an average of 7 months and had received antipsychotic medication.^48–50^ However, it is unlikely that the structural and functional alterations observed in the nAVH group were driven by alterations specifically associated with the onset of the illness or exposure to antipsychotic medication, since (1) the same number of FEP participants were allocated to the 2 AVH and nAVH groups; (2) these groups did not differ in terms of either severity of symptoms (with the exception of AVHs) or medication intake; (3) structural and functional connectivity values were not significantly associated with medication dose. In addition, the clinical heterogeneity associated with the ARMS status might have potentially impacted on our findings. For instance, ARMS individuals included in the nAVH group might have presented with a more severe impairment of frontotemporal connectivity because they had a higher risk to further develop psychosis. It seems unlikely that this was the case since frontotemporal connectivity alterations were no detected in the AVH group where a considerable number of FEP patients, who had therefore developed psychosis, were also included. At present, in addition, there is no robust evidence suggesting that alterations of frontotemporal connectivity represent a specific, reliable biomarker of transition to psychosis (see Pantelis et al^51^ and Fusar-Poli et al^52^ for detailed reviews). Nevertheless, given the cross-sectional nature of this study, we cannot exclude a potential impact of ARMS heterogeneity on our findings and future longitudinal studies are needed in order to better address this hypothesis.

In conclusion, the nAVH group relative to the control group showed a reduction of both white matter integrity and functional connectivity along the frontotemporal pathway of the perisylvian network as well as a disruption of the normal structure−function relationship. In contrast, the AVH group showed intermediate values for all measures and did not differ significantly from either group. These findings provide support to the hypothesis that a certain degree of preserved frontotemporal connectivity can be associated with the brain capability to generate AVHs in the early stage of the disorder.

## Supplementary Material

Supplementary material is available at http://schizophre niabulletin.oxfordjournals.org.

## Funding

Project Grant from the Wellcome Trust (WT085390/Z/08/Z). 

## Supplementary Material

Supplementary Data
