# Supplementary material for: Auditory Verbal Hallucinations and Brain Dysconnectivity in the Perisylvian Language Network: A Multimodal Investigation
Source: Schizophr Bull. 2013 Dec 22;41(1):192–200. doi: 10.1093/schbul/sbt172 (PMC4266279; doi:10.1093/schbul/sbt172)
Supplement: Supplementary Data [file supp_41_1_192__index.html]

Auditory Verbal Hallucinations and Brain Dysconnectivity in the Perisylvian Language Network: A Multimodal Investigation — Auditory Verbal Hallucinations and Brain Dysconnectivity in the Perisylvian Language Network: A Multimodal Investigation — Supplementary Data 

# Auditory Verbal Hallucinations and Brain Dysconnectivity in the Perisylvian Language Network: A Multimodal Investigation

## Supplementary Data

Data files

**Files in this Data Supplement:**

- Supplementary Data - Supplementary Data
