# Supplementary material for: Auditory Verbal Hallucinations and Brain Dysconnectivity in the Perisylvian Language Network: A Multimodal Investigation
Source: Schizophr Bull. 2013 Dec 22;41(1):192–200. doi: 10.1093/schbul/sbt172 (PMC4266279; doi:10.1093/schbul/sbt172)
Supplement: Supplementary Data [file supp_sbt172_Benetti_Mechelli_AVH_Duysconnectivity_SchBul_Supplementary.doc]

**Supplementary material**

Table 3. Coordinates and Z-scores (voxel-level P<0.05, FWE corrected) for regions activated during Initiation and Suppression relative to Repetition.

| ***Region*** | ***x*** | ***y*** | ***z*** | ***BA*** | ***Cluster size*** | ***Z score*** |
| --- | --- | --- | --- | --- | --- | --- |
| *Initiation & Suppression>Repetition* |  |  |  |  |  |  |
| L Medial Superior frontal gyrus | 0 | 12 | 62 | 6 | 1918 | 8.69 |
| L Inferior frontal gyrus | -50 | 14 | 30 | 45 | 3068 | 8.44 |
| L Middle temporal gyrus | -60 | -32 | -2 | 21 | 549 | 8.08 |
| R Inferior frontal gyrus | 42 | 24 | -8 | 47 | 270 | 8.01 |
| L Middle temporal gyrus | 30 | -32 | -4 | 21 | 151 | 7.32 |
|  |  |  |  |  |  |  |

Abbreviations: BA, Brodmann Area.
